# Benzoic acid fermentation from starch and cellulose via a plant-like β-oxidation pathway in *Streptomyces maritimus*

**DOI:** 10.1186/1475-2859-11-49

**Published:** 2012-04-30

**Authors:** Shuhei Noda, Eiichi Kitazono, Tsutomu Tanaka, Chiaki Ogino, Akihiko Kondo

**Affiliations:** 1 Department of Chemical Science and Engineering, Graduate School of Engineering, Kobe University, 1-1 Rokkodai, Nada, Kobe, 657-8501, Japan; 2 TEIJIN Holdings USA Inc, 165 Topaz Street Milpitas, New York,, CA, 95035, USA

**Keywords:** Streptomyces, Benzoic acid, Endo-glucanase, Cellulose

## Abstract

**Background:**

Benzoic acid is one of the most useful aromatic compounds. Despite its versatility and simple structure, benzoic acid production using microbes has not been reported previously. *Streptomyces* are aerobic, Gram-positive, mycelia-forming soil bacteria, and are known to produce various kinds of antibiotics composed of many aromatic residues. *S. maritimus* possess a complex amino acid modification pathway and can serve as a new platform microbe to produce aromatic building-block compounds. In this study, we carried out benzoate fermentation using *S. maritimus*. In order to enhance benzoate productivity using cellulose as the carbon source, we constructed endo-glucanase secreting *S. maritimus*.

**Results:**

After 4 days of cultivation using glucose, cellobiose, or starch as a carbon source, the maximal level of benzoate reached 257, 337, and 460 mg/l, respectively. *S. maritimus* expressed β-glucosidase and high amylase-retaining activity compared to those of *S. lividans* and *S. coelicolor*. In addition, for effective benzoate production from cellulosic materials, we constructed endo-glucanase-secreting *S. maritimus*. This transformant efficiently degraded the phosphoric acid swollen cellulose (PASC) and then produced 125 mg/l benzoate.

**Conclusions:**

Wild-type *S. maritimus* produce benzoate via a plant-like β-oxidation pathway and can assimilate various carbon sources for benzoate production. In order to encourage cellulose degradation and improve benzoate productivity from cellulose, we constructed endo-glucanase-secreting *S. maritimus.* Using this transformant, we also demonstrated the direct fermentation of benzoate from cellulose. To achieve further benzoate productivity, the L-phenylalanine availability needs to be improved in future.

## Background

In the past few decades, chemicals and fuel production from renewable resources have attracted attention due to global warming and limited supplies of fossil fuels [1-3]. The aromatic series include a large number of industrially important materials, and production of aromatic compounds using microorganisms is an active research area, as well as production of bio-fuel and other building-block compounds [4]. Phenol production using *Pseudomonas putida* and *p*-hydroxy cinnamic acid production using *P. putida* and *Escherichia coli* have been successfully demonstrated [5-7].

Benzoic acid is one of the most useful aromatic compounds, and can be converted to terephthalic acid by the Henkel reaction [8], epsilon-caprolactam by the Snia Viscosa process [9], and phenol by a decarbonation reaction [10]. Terephthalic acid is used to make polyethylene terephthalate and aramid, epsilon-caprolactam is a main component of nylon 6, and phenol is used to make polycarbonate. Benzoic acid is chemically synthesized via an oxidation reaction of toluene in the presence of potassium permanganate; however, this process is energy intensive. Despite its versatility and simple structure, benzoic acid production using microbes has not been reported previously.

**Figure 1:**
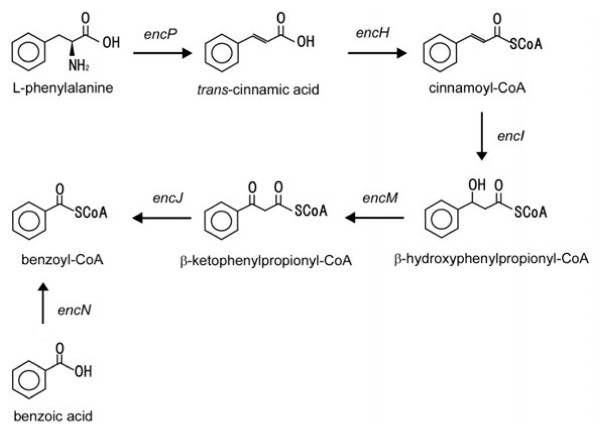
Proposed biosynthesis pathway from L-phenylalanine to benzoic acid.

*Streptomyces* are aerobic, Gram-positive, mycelia-forming soil bacteria, and are known to produce various kinds of antibiotics composed of many aromatic residues [11,12]. Piel et al. characterized the biosynthesis pathway of the polyketide bacteriostatic agent enterocin in the sediment-derived bacterium, *Streptomyces maritimus*[13]. *S. maritimus* possess a complex amino acid modification pathway and can serve as a new platform microbe to produce aromatic building-block compounds. Through a process involving β-oxidation of cinnamoyl-CoA into benzoyl-CoA, *S. maritimus* produce benzoyl-CoA in a plant-like manner from L-phenylalanine during the biosynthesis of the polyketide (Figure 1) [14]. *S. maritimus* is known to convert benzoic acid to polyketide via benzoyl-CoA using the gene encoding benzoyl-CoA ligase [15]. However, the conversion of benzoyl-CoA to benzoic acid has not been reveled in *S. maritimus.*

In the present study, we identified *S. maritimus* as a benzoate producer and succeeded in benzoate fermentation using a plant-like β-oxidation pathway in *S. maritimus*. Bio-production using a biomass resource with *Streptomyces* as a host has been an area of recent focus due to its great advantages in biomass assimilation [16-18]. In order to examine the carbon-assimilating ability of *S. maritimus* and apply it to benzoate fermentation, we used various carbon sources. We succeeded in benzoate fermentation using glucose, cellobiose, and starch. We compared the carbon-assimilating ability of *S. maritimus* with that of other model *Streptomyces*, and the versatility of *S. maritimus* as a host strain was also demonstrated. In benzoate fermentation from cellulose using wild-type *S. maritimus*, the amount of produced benzoate was considerably low, compared to using other carbon sources. The cellulose degradation to glucose and cello-oligosaccharide is one of rate-limiting steps. Here, to enhance benzoate productivity from cellulose, we constructed endo-glucanase (EG)-secreting *S. maritimus*. The engineered *S. maritimus* expressed EG from *Thermobifida fusca* YX and efficiently degraded cellulose. Using this strain, we successfully demonstrated the direct fermentation of benzoate cellulose.

## Results and discussion

### Identification and fermentation of benzoate using *S. maritimus*

**Figure 2:**
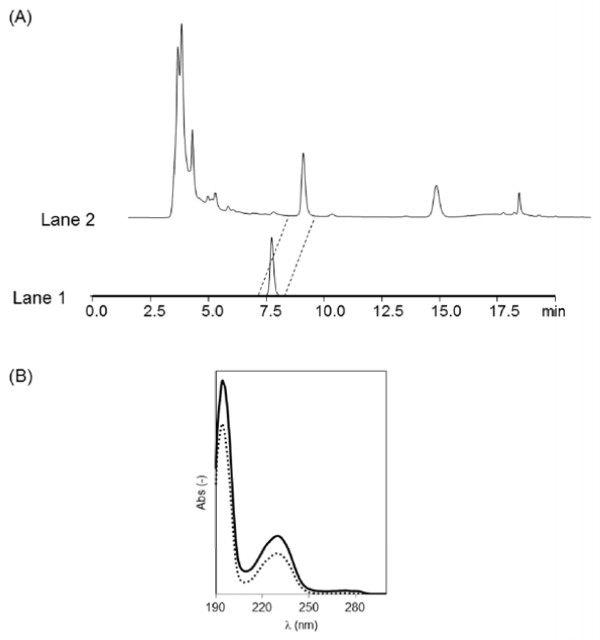
**(A) HPLC traces of benzoic acid analysis.** Lane 1: Standard sample of benzoic acid in acetonitrile: phosphate buffer (50 mM, pH 2.5) (30:70) solution. Lane 2: The culture supernatant of *S. maritimus*. **(B)** UV spectra of benzoic acid. 2.5 mg/l Standard sample of benzoic acid in acetonitrile: phosphate buffer (50 mM, pH 2.5) (30:70) solution (dotted line), 4 mg/l benzoic acid fraction separated from the culture supernatant of *S. maritimus* by HPLC (solid line).

After cultivation of *S. maritimus*/WT using TSB medium, benzoate produced in *S. maritimus* was identified using a co-chromatography method and UV spectrophotometer [19,20]. Figure 2(A) shows a chromatogram of a standard sample of benzoic acid solution (Lane 1) and the culture supernatant of *S. maritimus*/WT containing produced benzoic acid (Lane 2). The peak of benzoic acid in the standard sample was found at about 8 min (Lane 1), which was also observed in the culture supernatant of *S. maritimus*/WT (Lane 2). The addition of L-phenylalanine into the initial culture medium increased the peak areas of benzoic acid (data not shown). The UV spectra of the benzoic acid fraction separated from the culture supernatant of *S. maritimus*/WT exhibit two major absorption peaks in the region of 190–300 nm, similar to the standard sample of benzoic acid (Figure 2(B)). In addition, we carried out MS spectra analysis. MS and tandem MS analysis show that the peak of benzoic acid dehydrogenated and decarboxylated was observed around m/z 121.10 and 77.00, respectively. These results demonstrated that *S. maritimus*/WT produces benzoate in the culture supernatant.

**Figure 3:**
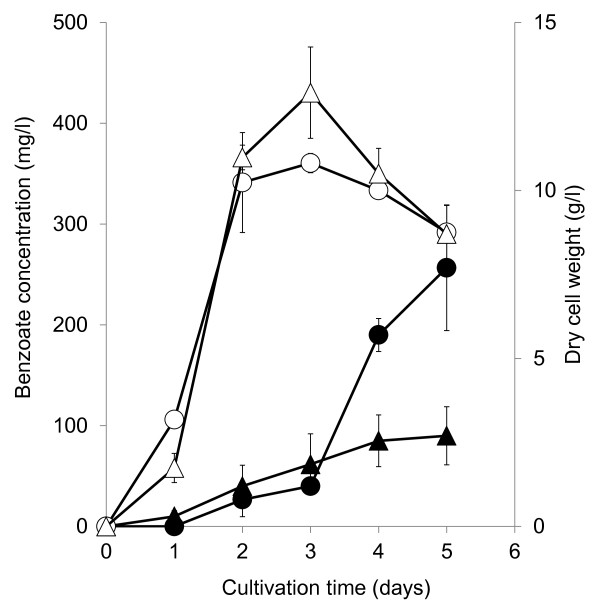
**Time-courses of dry cell weight using glucose as the sole carbon source:* S. maritimus*/WT in modified TSB medium with 5% tryptone and 3% glucose (open circles);* S. maritimus*/WT in modified TSB medium with 5% tryptone and 3% xylose (open triangles).** Time-courses of produced benzoate in culture: *S. maritimus*/WT in modified TSB medium with 5% tryptone and 3% glucose (closed circles); *S. maritimus*/WT in modified TSB medium with 5% tryptone and 3% xylose (closed triangles). The dry cell weight and benzoate concentration were determined in the same culture. Each data point shows the average of three independent experiments, and error bars represent standard deviation.

In order to enhance benzoate productivity, we tested benzoate fermentation using 3% glucose or xylose as the carbon source using *S. maritimus*/WT. Figure 3(A) shows time-courses of dry cell weight. *S. maritimus*/WT consumed glucose or xylose within 3 days (data not shown), and the maximum dry cell weight of *S. maritimus*/WT using xylose was slightly higher than that using glucose (Figure 3(A)). Figure 3(B) shows the amount of produced benzoate. Although it had similar cell-growth ability, the maximal amount of produced benzoate from glucose was 260 mg/l, which was 3-fold higher than that using xylose (Figure 3(B)). Figure 3 shows benzoate production started after cell growth reached the maximal level. This indicates that the cell growth before benzoate production is one of key factor in benzoate fermentation using *S .maritimus*. In this study, *S. maritimus* completely consumed 3% glucose after 3 days cultivation, and the cell of *S. maritimus* reached the maximal level after 3 days. The cell growth is a key factor of benzoate fermentation using *S. maritimus*. These imply that sugar uptake is one of rate-limiting step in cell growth and benzoate fermentation using *S. maritimus*. In *Streptomyces*, a lot of the genes encoding sugar transporter were identified. The sugar consumption may be improved by introducing those genes, and the more rapid cell growth and production of benzoate may be achieved. The addition of L-phenylalanine improved benzoate productivity from xylose (data not shown), which indicates that L-phenylalanine availability in *S. maritimus* using xylose as the carbon source is not enough for benzoate production.

In enterocin biosynthesis pathway of *S. maritimus*, benzoyl-CoA, which is a precursor of benzoate, is converted to enterocin by multiple enzymes. Here, to improve benzoate productivity, we carried out inactivation of *encABCL* closely concerning enterocin production. We successfully disrupted *encABCL* in *S. maritimus*, and the engineered strain was named *S. maritimus*/Δ*encABCL*. Using *S. maritimus*/Δ*encABCL*, benzoic acid fermentation was tested. Inactivation of *encABCL* had no negative effect on the cell growth of *S. maritimus*, however, benzoic acid productivity drastically decreased, compared to wild-type *S. maritimus* (data not shown).

### Benzoate production from cellobiose and starch using wild-type *S. maritimus*

Although some *Streptomyces* are known to assimilate various carbon sources, such as oligosaccharides and starch [18,21], the carbon-assimilating ability and growth profile of *S. maritimus* have not been investigated.

**Figure 4:**
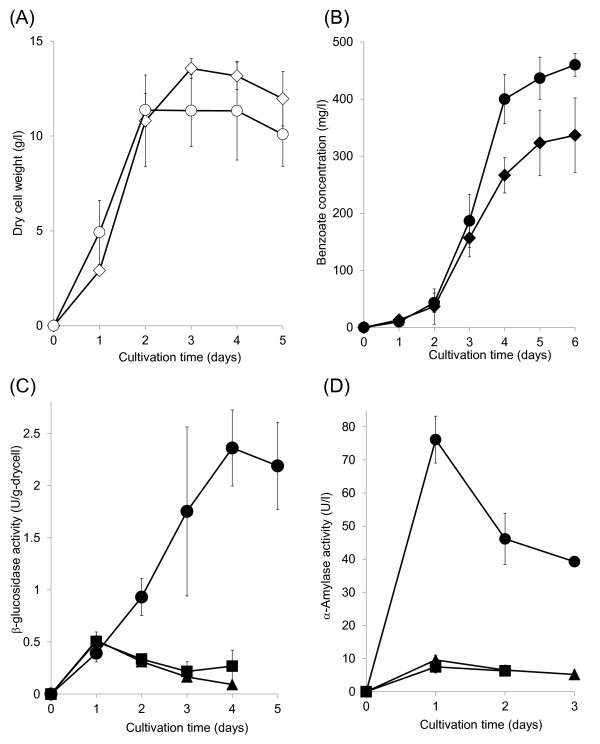
**(A) Time-courses of dry cell weight using glucose as the sole carbon source:*S. maritimus*/WT in modified TSB medium with 5% tryptone and 3% starch (open circles);*S. maritimus*/WT in modified TSB medium with 5% tryptone and 3% cellobiose (open diamonds). (B)** Time-courses of produced benzoate in culture: *S. maritimus*/WT in modified TSB medium with 5% tryptone and 3% starch (closed circles); *S. maritimus*/WT in modified TSB medium with 5% tryptone and 3% cellobiose (closed diamonds). **(C)** Time-courses of β-glucosidase activity in the intracellular fractions of *S. maritimus*/WT, *S. lividans*, and *S. coelicolor* using modified TSB medium with 5% tryptone and 3% cellobiose: *S. maritimus*/WT (closed circles), *S. lividans* (closed squares), *S. coelicolor* (closed triangles). **(D)** Time-courses of α-amylase activity in the culture supernatant of *S. maritimus*/WT, *S. lividans*, and *S. coelicolor* modified TSB medium with 5% tryptone and 3% starch: *S. maritimus*/WT (closed circles), *S. lividans* (closed squares), *S. coelicolor* (closed triangles). The dry cell weight and benzoate concentration were determined in the same culture. Each data point shows the average of three independent experiments, and error bars represent standard deviation.

Figure 4(A) shows the time-course of dry cell weight using 3% cellobiose, and the maximum dry cell weight of *S. maritimus*/WT using cellobiose is higher than that using glucose. As shown in Figure 4(B), the maximal level of produced benzoate using 3% cellobiose was 323 mg/l after 6 days of cultivation, and the estimated yield for benzoate was 1.76 Cmol% (Table 1). In the present study, *S. maritimus*/WT almost completely consumed 3% cellobiose within 2 days (data not shown). We compared the β-glucosidase (BGL) activity of *S. maritimus* to that of *S. lividans* and *S. coelicolor*, which are model strains in *Streptomyces*. Figure 4(C) shows the time-courses of BGL activity in the intracellular fractions of *S. maritimus, S. lividans*, and *S. coelicolor*. The intracellular BGL activity detected in *S. maritimus* reached 46.1 U/g-drycell, which was 5-fold higher than that in *S. lividans* and *S. coelicolor* (Figure 4(C)) after 4 days of cultivation. Although the BGL activity expressed in *S. maritimus* was vastly higher than those in *S. lividans* or *S. coelicolor*, the cellobiose consumption rates among these strains were almost the same. This result indicates that cellobiose uptake is the rate-limiting step, and that overexpression of the sugar transporter may improve the cellobiose consumption and benzoate productivity in *S. maritimus*. Although the benzoate productivity using xylo-oligosaccharide was lower, similar to that using xylose, the cell growth was similar to that using other carbon sources (data not shown).

**Table 1:** **Various parameters in benzoate fermentation**^***a***^

Strain	Carbon source	Maximum dry cell weight (g/l)	Benzoate produced (mg/l)	Yield^*b*^ (Cmol%)
*S. maritimus*/WT	Glucose (3%)	10.8 ± 0.29	257 ± 62.4	1.47
*S. maritimus*/WT	Xylose (3%)	12.9 ± 1.36	90.0 ± 28.9	0.52
*S. maritimus*/WT	Cellobiose (3%)	13.6 ± 0.52	337 ± 65.5	1.83
*S. maritimus*/WT	Corn starch (3%)	11.4 ± 0.88	460 ± 36.8	2.64
*S. maritimus*/WT	PASC (1%)	-	23.3 ± 8.17	0.40
*S. maritimus*/*ps-tfu0901*	PASC (1%)	-	125 ± 8.96	2.15
*S. maritimus*/*ps-tfu1074*	PASC (1%)	-	103 ± 0.94	1.77

^*a*^ Values represent means ± standard deviation for three independent experiments.

^*b*^ mol of carbon involved in produced benzoate per mol of carbon involved in consumed carbon source.

We carried out benzoate fermentation using starch. Figure 4(A) shows the time-course of dry cell weight using 3% starch. The cell growth of *S. maritimus* using starch as the carbon source was similar to that using glucose (Figure 3(A), 4(A)). As shown in Figure 4(B), the maximal level of produced benzoate using 3% starch was 460 mg/l after 6 days of cultivation. The estimated yield of benzoate using starch as the carbon source was 2.64 Cmol%, which was 1.77 times higher than that using glucose (Table 1). Surprisingly, benzoate production using starch as the carbon source was more efficient than when using glucose. This indicates that *S. maritimus* can directly assimilate starch for benzoate production more effectively than glucose. Although the cell growth of *S. maritimus* using cellobiose was higher than that of using starch, the maximal level of produced benzoate using starch was about 1.5 times greater, compared to using cellobiose. This may indicate that starch as the carbon source encourages the carbon flux to flows smoothly to benzoate synthesis pathway in *S. maritimus*. Here, to demonstrate that additional carbon sources were used for benzoate formation and the cell growth of *S. maritimus*, we carried out benzoate fermentation in *S. maritimus* using TSB medium with additional 5% tryptone (without additional each carbon source). The maximal level of produced benzoate reached about only 100 mg/L, and the cell growth of *S. maritimus* was drastically low, compared to fermentation with additional carbon source. Hence, we conclude that additional glucose, cellobiose, and starch were used for the cell growth, and encouraged benzoate formation.

After the fermentation, the amount of produced cinnamic acid, one of the easily detected intermediates, was 170 mg/l (from 3% starch) and 73 mg/l (from 3% glucose), respectively. These results show that the ratio of cinnamic acid to benzoic acid (mol/mol) was increased along with the increased the amount of produced benzoate, suggesting that optimization of the carbon flux may improve benzoate productivity. Enhancing L-phenylalanine availability in the cell may also lead to further benzoate productivity, because the amount of produced benzoate in fermentation using TSB medium with 1.5% glucose, 1.5% tryptone, and additional 100 mM of L-phenylalanine reached 1.3 g/L. The biosynthesis pathway of L-phenylalanine is strictly regulated by feedback inhibition concerning produced aromatic amino acids. The random mutagenesis by means of *N*-methyl-*N*’-nitro-*N*-nitrosoguanidine (NTG) treatment, followed by selection on solid medium containing phenylalanine analogs, can be effective way to obtain the mutants that be insensitive to feedback inhibition. In addition, one transcriptional activator and two regulator proteins have been identified in the polyketide synthesis gene cluster in *S. maritimus* involving genes concerning benzoate production [13]. Overexpression or deletion of those genes may also enhance the yield of benzoate. Figure 4(D) shows the time-courses of α-amylase (AMY) activity in the culture supernatant of *S. maritimus, S. lividans*, and *S. coelicolor*. The AMY activity in the culture supernatant of *S. maritimus* after 2 days of cultivation is more than 8-times higher than that of *S. lividans* and *S. coelicolor*.

*S. lividans* and *S. coelicolor* are known as model *Streptomyces*. The carbon source metabolite pathways and genes concerning various biomass degradation enzymes in *S. lividans* and *S. coelicolor* have been widely studied [21,22], and they have been used as production hosts for enzymes and chemical compounds [16-18,25]. In this study, we used *S. maritimus* for benzoate production using various carbon sources and demonstrated that *S. maritimus* expresses high BGL- and AMY-retaining activity compared to that of *S. lividans* and *S. coelicolor*. Our results may indicate that *S. maritimus* is a new candidate host strain for useful compound production using biomass resources.

### Benzoate production from cellulosic materials using EG-secreting *S. maritimus*

Many microbes have difficulty degrading cellulose due to its rigid structure. Although we investigated the cellulose-assimilating ability of *S. maritimus*/WT, the EG activity expressed by *S. maritimus*/WT was not enough to degrade cellulose sufficiently.

**Figure 5:**
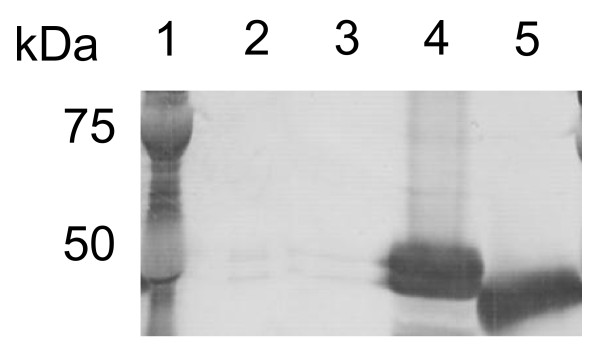
**Western blot analysis of Tfu0901-(His)**_**6**_** and Tfu1074-(His)**_**6**_**.** Lane 1: Protein marker; Lane 2: *S. lividans*/WT; Lane 3: *S. lividans*/pTONA4; Lane 4: *S. maritimus*/*ps*-*tfu0901*; Lane 5: *S. maritimus*/*ps*-*tfu1074.*

For effective benzoate production from cellulosic materials, we selected two candidate EG, Tfu0901 and Tfu1074, from *T. fusca* YX [23]. The genes encoding Tfu0901 or Tfu1074 were introduced downstream of the phospholipase D promoter region from *Streptoverticillium cinnamoneum* in the multi-copy type vector pTONA4. For effective secretion of EG, signal peptide sequence from phospholipase D from *Stv. cinnamoneum* (*pld-s*) was fused to upstream of those two EG genes, and Tfu0901 or Tfu1074 in over-expressed in *S. maritimus*, respectively. The supernatants of *S. maritimus*/*pstfu0901* and *S. maritimus*/*pstfu1074* after 3 days of cultivation were analyzed by western blotting. A band corresponding to Tfu0901 (calculated molecular mass, 46 kDa) or Tfu1074 (calculated molecular mass, 43 kDa) was clearly observed (Figure 5, lanes 4 and 5), whereas no band was observed in the case of *S. lividans*/WT (Figure 5, lane 2) or *S. lividans*/pTONA4 (Figure 5, lane 3). These results show successful secretory expression of Tfu0901 and Tfu1074 using *S. maritimus*. We demonstrated that *pld-s* encourages protein secretion in *S. maritimus*, as well as *S. lividans*.

**Figure 6:**
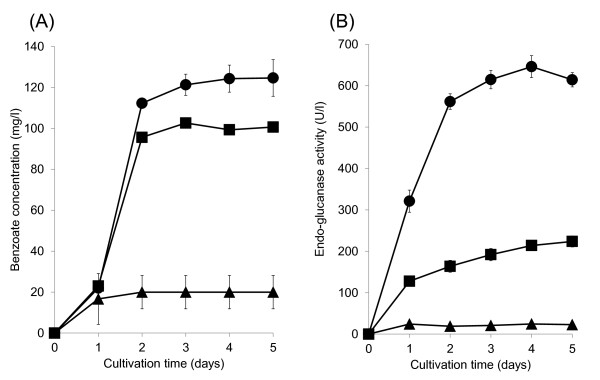
**(A) Time-courses of produced benzoate in culture:*S. maritimus*/*ps*-*tfu1074*in modified TSB medium with 5% tryptone and 1% phosphoric acid swollen cellulose (PASC) (closed circles);*S. maritimus*/*ps*-*tfu0901* in modified TSB medium with 5% tryptone and 1% PASC (closed diamonds);*S. maritimus*/WT in modified TSB medium with 5% tryptone and 1% PASC (closed triangles). (B)** Time-courses of endo-glucanase activity in the culture supernatant: *S. maritimus*/*ps*-*tfu1074* in modified TSB medium with 5% tryptone and 1% PASC (closed circles); *S. maritimus*/*ps*-*tfu0901* in modified TSB medium with 5% tryptone and 1% PASC (closed diamonds); *S. maritimus*/WT in modified TSB medium with 5% tryptone and 1% PASC (closed triangles). Each data point shows the average of three independent experiments, and error bars represent standard deviation.

Using *S. maritimus*/WT, *S. maritimus*/*ps*-*tfu0901*, and *S. maritimus*/*ps*-*tfu1074* strains, we carried out benzoate fermentation from phosphoric acid swollen cellulose (PASC). Figure 6(A) shows the time-course of benzoate production using 1% PASC as the carbon source. The maximal level of produced benzoate was 125 or 103 mg/l after 4 days of cultivation using *S. maritimus*/*ps*-*tfu0901* or *S. maritimus*/*ps*-*tfu1074*, respectively, whereas *S. maritimus*/WT produced 23.3 mg/l benzoate. Figure 6(B) shows the time-course of EG activity using 1% PASC as the carbon source. The maximum EG activities of *S. maritimus*/*ps*-*tfu0901* and *S. maritimus*/*ps*-*tfu1074* were 646 and 224 U/l (Figure 6(B)), respectively. EG activity detected in the culture supernatant of *S. maritimus*/*ps*-*tfu0901* was 3-fold higher than that of *S. maritimus*/*ps*-*tfu1074*. The higher activity of Tfu0901 may be attributed to the large amount of expressed Tfu0901, which was confirmed by SDS-PAGE analysis of the culture supernatant (data not shown). The estimated yields of benzoate from 1% PASC using *S. maritimus*/*ps*-*tfu0901* or *S. maritimus*/*ps*-*tfu1074* were 2.15 and 1.77 Cmol%, which were 5.4- and 4.4-times higher than that using *S. maritimus*/WT, respectively (Table 1).

## Conclusions

We examined the carbon-assimilating ability of *S. maritimus*, which was greater than other model *Streptomyces*, and successfully demonstrated direct benzoate fermentation from starch using wild-type *S. maritimus* and cellulose using genetically modified *S. maritimus*. EG-secreting *S. maritimus* efficiently produced benzoate using cellulose just as well as when using glucose. This is the first report concerning benzoate production from cellulose or starch using microbes.

## Methods

### Strain and medium

Wild-type *S. maritimus* (*S. maritimus*/WT) was used as the host for benzoate fermentation. For the production of benzoate, a single colony of *S. maritimus*/WT was inoculated in a test tube containing 5 ml of TSB medium [1.7% pancreatic digest of casein, 0.3% papaic digest of soybean meal, 0.25% glucose, 0.5% sodium chloride, and 0.25% dipotassium phosphate (BD Diagnostic Systems, Sparks, MD, USA)] supplemented with 50 μg/ml of kanamycin, followed by cultivation at 28°C for 3 days. Then, 5 ml of the preculture medium of *S. maritimus*/WT was seeded into a shake flask with a baffle containing 100 ml of modified TSB medium with 5% tryptone, 50 μg/ml kanamycin, and one of either 3% glucose, 3% xylose, 3% cellobiose, or 3% cornstarch (Nacalai Tesque, Kyoto, Japan) as a carbon source, followed by incubation at 28°C for 5–6 days.

### Plasmid construction and transformation

*Escherichia coli* NovaBlue {*endA1 hsdR17*(r_K12_^-^ m_K12_^+^) *supE44 thi-IgyrA96 relA1lac recA1/F’*[*proAB*^+^*lacI*^q^ ZΔM15::Tn*10*(Tet^r^)]} (Novagen, Inc., Madison, WI, USA), used to construct plasmids, was grown in Luria-Bertani (LB) medium containing 100 or 40 μg/ml ampicillin or kanamycin at 37°C.

The vectors for protein expression using *S. maritimus* as a host and gene deletion of *S. maritimus* were constructed as follows. The strains and the plasmids used in this study are summarized in Table 2. Polymerase chain reaction (PCR) was carried out using PrimeSTAR HS (Takara, Shiga, Japan).

**Table 2:** Strains and plasmids used in this study

Strain, plasmid, or primer	Relevant features	Source or reference
**Strains**		
*Escherichia coli* strains		
Nova blue	*endA1 hsdR17*(r_*K12*_^*-*^m_*K12*_^+^) *supE44 thi-I gyrA96 relA1 lac recA1/F’*[*proAB*^+^*lacI*^q^ ZΔM15::Tn*10*(Tet^r^)]	Novagen
S17-1 λpir	*TpR SmRrecA*, *thi, pro*, *hsdR*-M^+^RP4: 2-Tc:Mu: Km Tn*7* λpir	BIOMEDAL
*Streptomyces maritimus*		
/WT	DSMZ 41777 WT strain	DSMZ
/*ps-tfu0901*	DSMZ 41777 strain with *tfu0901*-secreting expression vector	This study
/*ps-tfu1074*	DSMZ 41777 strain with *tfu1074*-secreting expression vector	This study
		This study
*Streptomyces lividans*1326	WT strain	NBRC
*Streptomyces coelicolor*A3(2)	WT strain	NBRC
**Plasmids**		
pUC702-prom-sig-term	Versatile vector for protein expression; thiostrepton resistance marker; *pld* promoter; *rep*, replication gene from pIJ101; size, 8,600 bp	25
pUC702-*ps*-Tfu0901-(His)_6_	Vector for secreting endoglucanase (Tfu0901); thiostrepton resistance marker; *pld* promoter; *rep*, replication gene from pIJ101; size, 9,900 bp	This study
pUC702-*ps*-Tfu1074-(His)_6_	Vector for secreting endoglucanase (Tfu1074); thiostrepton resistance marker; *pld* promoter; *rep*, replication gene from pIJ101; size, 9,800 bp	25
pTONA4	Versatile vector for protein expression; kanamycin and thiostrepton resistance marker; *pld* promoter; *rep*, replication gene from pIJ101; size, 9,000 bp	This study
pTONA4-*ps-tfu0901*	Vector for secreting endoglucanase (Tfu0901); kanamycin and thiostrepton resistance marker; *pld* promoter; *rep*, replication gene from pIJ101; size, 10,300 bp	This study
pTONA4-*ps-tfu1074*	Vector for secreting endoglucanase (Tfu1074); kanamycin and thiostrepton resistance marker; *pld* promoter; *rep*, replication gene from pIJ101; size, 10,200 bp	This study

The promoter and terminator regions of pTONA5 were replaced with Phospholipase D promoter and terminator regions from *Streptoverticillium cinnamoneum*. The vector was called pTONA4 [24].

The expression plasmids for His-tagged Tfu0901 (Tfu0901-(His)_6_) and Tfu1074 (Tfu1074-(His)_6_) were constructed as follows. Tfu0901 was amplified by PCR using the *Thermobifida fusca* YX genome as a template with the following primers: 5’-AAGCTAGCGGTCTCACCGCCACAGTCACCAAAG-3’ (*Nhe*I-Tfu0901/Fw) and 5’-TGGATCCTCAGTGGTGGTGGTGGTGGTGGGACTGGAGCTTGCTCCGCACC-3’ (*Bam*HI-Tfu0901/Rv). The amplified fragments were digested with *Nhe*I and *Bam*HI and introduced into the *Nhe*I and *Bam*HI sites of pUC702-prom-sig-term [25]. The resultant plasmids were called pUC702-*ps*-Tfu0901-(His)_6_. The fragments of *ps*-Tfu0901-(His)_6_ and *ps*-Tfu1074-(His)_6_ were amplified by PCR using pUC702-*ps*-Tfu0901-(His)_6_ and pUC702-*ps*-Tfu1074-(His)_6_ as a template with the following primers: 5’-TCGTTTAAGGATGCAGCATGCTCCGCCACCGGCTCCGCCG-3’ and 5’-CGCTCAGTCGTCTCAGTGGTGGTGGTGGTGGTGGGACTGGAGCTTGCT-3’ or 5’-TCGTTTAAGGATGCAGCATGCTCCGCCACCGGCTCCGCCG-3’ and 5’-CGCTCAGTCGTCTCAGTGGTGGTGGTGGTGGTGGCTGGCGGCGCAGGT-3’, respectively. Each of the amplified fragments was introduced into the *Nde*I and *Hind*III sites of pTONA4 with In-Fusion HD Cloning kit (Takara), respectively [25]. The resultant plasmids were called pTONA4-*pstfu0901* and pTONA4-*pstfu1074*, respectively.

### Intergeneric conjugation and cultivation

*E. coli* S17-1 λpir (*TpR SmRrecA*, *thi, pro*, *hsdR*-M^+^RP4: 2-Tc:Mu: Km Tn*7* λpir) was transformed with each constructed plasmid. A single colony of each transformant was cultivated in 3 ml of LB medium containing 40 μg/ml kanamycin at 37°C for 8 h. Cells were harvested, and the cell suspension was washed three times with LB broth and centrifuged to remove kanamycin. The cells were then suspended in 500 μl of LB broth and mixed with *S. maritimus* spores. The mixture was plated on ISP4 medium (1.0% soluble starch, 0.1% K_2_HPO_4_, 0.1% MgSO_4_·7H_2_O, 0.1% NaCl, 0.2% (NH_4_)_2_SO_4_, 0.2% CaCO_3_, 0.0001% FeSO_4_, 0.0001% MnCl_2_, 0.0001% ZnSO_4_, and 2.0% agar). The mixture was then incubated for 18 h at 30°C. A 3-ml aliquot of soft-agar nutrient broth containing kanamycin (50 μg/ml) and nalidixic acid (67 μg/ml) was dispensed in layers on the plate, which was then incubated at 30°C for 5–7 days. A single colony was streaked on an ISP4 agar plate containing kanamycin (50 μg/ml) and nalidixic acid (5 μg/ml). The plate was incubated at 30°C for 5–7 days, and selected transformants were named *S. maritimus*/pTONA4, *S. maritimus*/*ps*-*tfu0901* and *S. lividans*/*ps*-*tfu1074*, respectively.

For production of benzoate, a single colony of *S. maritimus*/*ps*-*tfu0901* and *S. maritimus*/*ps*-*tfu1074* were inoculated in a test tube containing 5 ml of TSB medium supplemented with 50 μg/ml of kanamycin, followed by cultivation at 28°C for 3 days. Then, 5 ml of the preculture medium of *S. maritimus*/*ps*-*tfu0901* and *S. maritimus*/*ps*-*tfu1074* were seeded into a shake flask with a baffle containing 100 ml of modified TSB medium with 5% tryptone, 50 μg/ml kanamycin, and 1% phosphoric acid swollen cellulose (PASC) as a carbon source, followed by incubation at 28°C for 5–6 days.

### Analytical methods

The benzoic acid and cinnamic acid concentration was simultaneously determined by high-performance liquid chromatography (HPLC; Shimadzu, Kyoto, Japan) using a Cholester column (Cholester residues, 5 μm, 4.6 × 250 mm, Nacalai Tesque). The operating conditions were 30°C, with a flow rate of 1.2 ml/min. A dual solvent system was used. Solvent A was phosphate buffer (50 mM, pH 2.5) and solvent B acetonitrile. The gradient started at 70% of solvent A and 30% of solvent B, A 50–50 mixture of A and B was used from 12 to 17 min. A 70–30 mixture of A and B was used from 17.01 to 20.00 min. The peak of benzoic acid and cinnamic acid in the standard sample were found at about 8 and 12.5 min, respectively. Then, the benzoic acid and cinnamic acid concentration was determined using an ultra-violet absorbance detector (SPD-20AV, Shimadzu). The culture supernatant was separated from the culture broth by centrifugation at 21,880 × *g* for 20 min, which was followed by analysis using HPLC. UV absorption spectra were obtained using a JASCO V-650 spectrophotometer (JASCO Corporation, Tokyo, Japan). Mass and tandem mass spectra were acquired using 6460 triple stage mass spectrometer (Agilent 6460 with Jet Stream Technology, Agilent Technologies,Waldbronn, Germany) operated at negative ion mode. Voltages for the collision energy and fragmentor voltage were set at 5 and 60 V, respectively. Spectroscopic data of benzoic acid: UV λ_max_^MeOH^ nm: 280; ESI-MS *m/z*: 121.1 ([M-H]^-^); ESI-MS/MS *m/z* (relative intensity(%)) 121.1(65, [M-H]^-^), 77.0 (100, [M-CO_2_-H]^-^).

### Measurement of biomass degradation enzyme activity

β-Glucosidase activity was measured in 25 μl of 1 M Tris–HCl buffer (pH 7.0) with 100 μl of 10 mM *p*-nitrophenyl-β-D-glucopyranoside (*p*NPG) (Nacalai Tesque) as the substrate. The mixture (containing 375 μl of culture supernatant diluted to 10%) was incubated at 30°C for 60 min. The reaction was terminated by the addition of 500 μl of 3 M sodium carbonate, and the *p*-nitrophenol released was determined by measuring absorbance at 400 nm. One unit of enzyme activity was defined as the amount of enzyme that released 1 μmol of *p*-nitrophenol from the substrate per min.

Amylase and EG activity were measured according to a method established by Miller [16], with some modification. A 300-μl aliquot of culture supernatant and cell fractions was mixed with 700 μl of a 1% (w/v) solution of raw starch, which was then dissolved in either 100 mM Tris–HCl buffer (pH 7.0) or carboxy methyl cellulose (CMC) dissolved in 100 mM Tris–HCl buffer (pH 7.0). The mixture was incubated at 37 and 50°C for 4 and 1 h, respectively. The amount of reducing sugar released from starch and CMC was assayed by determining the amount of glucose, and equivalents, using the dinitrosalicylic acid method [16]. One unit of enzyme activity was defined as the amount of enzyme that released 1 μmol of reducing sugar as glucose, for starch and CMC, and equivalents, from the substrate per min.

### Western blotting

*S. maritimus*/pTONA4, *S. maritimus*/*ps*-*tfu0901* and *S. lividans*/*ps*-*tfu1074* were cultured at 28°C for 3 day in 100 ml TSB medium with 3% cellobiose, 5% tryptone, and 50 μg/ml kanamycin, respectively. Sodium dodecyl sulfate-polyacrylamide gel electrophoresis (SDS-PAGE) loading buffer was added to the supernatant, followed by boiling at 100°C for 5 min. Proteins were analyzed by SDS-PAGE using an SDS-polyacrylamide gel (15%: w/v), after which proteins were electroblotted onto a polyvinylidene difluoride membrane (Millipore Co., Boston, MA, USA) and were allowed to react with primary rabbit anti-(His)_6_ and secondary goat anti-rabbit immunoglobulin G alkaline-phosphatase-conjugated antibodies (Promega Co., Madison, WI, USA). The membrane was then stained with nitroblue tetrazolium (Promega) and 5-bromo-4-chloro-3-indolylphosphate (Promega) according to the manufacturer’s protocol.

## Abbreviations

EG, Endo-glucanase; BGL, β-glucosidase; AMY, α-amylase; PASC, Phosphoric acid swollen cellulose.

## Competing interests

The authors declare that they have no competing interests.

## Authors’ contributions

S.N. and E.K. designed the experiments. S.N. performed the experiments. S.N. and T.T. wrote the paper. C.O. and A.K. commented and supervised on the manuscript. All authors approved the final manuscript.
